# A numerical framework for interstitial fluid pressure imaging in poroelastic MRE

**DOI:** 10.1371/journal.pone.0178521

**Published:** 2017-06-06

**Authors:** Likun Tan, Matthew D. J. McGarry, Elijah E. W. Van Houten, Ming Ji, Ligin Solamen, Wei Zeng, John B. Weaver, Keith D. Paulsen

**Affiliations:** 1 Thayer School of Engineering, Dartmouth College, Hanover, NH 03755, United States of America; 2 Department of Biomedical Engineering, Columbia University, New York, NY 10027, United States of America; 3 Department of Mechanical Engineering, University de Sherbrooke, Sherbrooke, Quebec J1K 2R1, Canada; 4 Department of Molecular Biology and Genetics, Johns Hopkins University School of Medicine, Baltimore, MD 21205, United States of America; 5 Department of Radiology, Dartmouth-Hitchcock Medical Center, Lebanon, NH 03756 United States of America; 6 Norris Cotton Cancer Center, Dartmouth-Hitchcock Medical Center, Lebanon, NH 03756 United States of America; Rensselaer Polytechnic Institute, UNITED STATES

## Abstract

A numerical framework for interstitial fluid pressure imaging (IFPI) in biphasic materials is investigated based on three-dimensional nonlinear finite element poroelastic inversion. The objective is to reconstruct the time-harmonic pore-pressure field from tissue excitation in addition to the elastic parameters commonly associated with magnetic resonance elastography (MRE). The unknown pressure boundary conditions (PBCs) are estimated using the available full-volume displacement data from MRE. A subzone-based nonlinear inversion (NLI) technique is then used to update mechanical and hydrodynamical properties, given the appropriate subzone PBCs, by solving a pressure forward problem (PFP). The algorithm was evaluated on a single-inclusion phantom in which the elastic property and hydraulic conductivity images were recovered. Pressure field and material property estimates had spatial distributions reflecting their true counterparts in the phantom geometry with RMS errors around 20% for cases with 5% noise, but degraded significantly in both spatial distribution and property values for noise levels > 10%. When both shear moduli and hydraulic conductivity were estimated along with the pressure field, property value error rates were as high as 58%, 85% and 32% for the three quantities, respectively, and their spatial distributions were more distorted. Opportunities for improving the algorithm are discussed.

## Introduction

Magnetic resonance elastography (MRE) is a noninvasive, quantitative imaging technique that characterizes material property distributions of biological tissues through application of appropriate constitutive models. Previous work has generally modeled biological tissue as a single solid matrix that is linearly elastic, isotropic, and nearly incompressible, and has focused on estimating the viscoelastic properties of breast [1–3], liver [4, 5], muscle [6–8], and prostate [9]. Recent work in MRE involves measurements of brain tissue whose behavior is inadequately described by linear elasticity. Instead, some soft tissues like the brain may be better represented as a biphasic material, in which fluid and solid phases coexist, and whose mechanical behavior can be approximated by a porous elastic matrix with an infiltrating pore fluid.

Experimental studies [10] have shown that brain tissue consists of a matrix of neurons and glial cells containing both intracellular and extracellular fluid. Approximately 20% of tissue volume consists of extracellular fluid which can move within the interstitium through the network of capillaries and the lymphatic system and plays the role of an infiltrating pore fluid from the modeling perspective. Furthermore, the network of neural and glial cells provides structural support that acts as the porous elastic matrix. Mechanical testing results under controlled drainage conditions also suggest the behavior of brain tissue is well described by a poroelastic model [11, 12].

Originally developed by Biot in 1956 [13, 14] for soil mechanics, the poroelastic model assumes volumetric deformation of the solid matrix leads to fluid flow in the material, and conversely, fluid forced into the material causes deformation of the matrix. The model was extended to time-harmonic behavior by Cheng *et al*. [15] and later by Perrinez *et al.* [16] to the frequency-domain equivalent set of equations for tissue elastography applications known as poroelastic MRE.

So far, poroelastic modeling of brain tissue using finite elements have been successful in capturing quasi-static deformation in hydrocephalus and edema [17, 18], as well as brain-shift and interstitial pressure fluctuations during stereotactic neurosurgery [19–23]. However, the time-harmonic pressure distribution that develops under the natural cerebrovascular pulsations at cardiac frequencies, which have been used as sources of motion in MRE (termed intrinsic actuation) [24], has not been investigated to date. The pressure field in this case may be interpreted as variation in the interstitial fluid pressure (IFP) of the tissue caused by the blood pressure pulse. Solid tumors typically show increased IFP [25–28] which is a barrier to the delivery of cancer therapy [29–32]. Elevated IFP of a tumor also indicates poor prognosis for both chemotherapy and radiation therapy [33–35], and cases which respond well to therapy often show a progressive decrease of IFP over the course of the treatment [36]. High IFP of primary tumor has also been linked to greater chances of recurrence and distant metastases [35, 37]. Moreover, compounds which lower IFP have been shown to increase the therapeutic benefit of traditional cancer therapies due to more efficient uptake of therapeutic agents [38, 39]. Elevated IFP in tumors causes even greater obstacles for large molecules (including the new generation of genetically engineered cancer therapies) because the dominant mechanism of transport is convection, whereas smaller molecules can also travel by diffusion, which is not as strongly affected by pressure [40]. *In vivo* measurements of IFP could provide valuable information for treatment planning and monitoring of solid tumors.

Currently, direct measurements of IFP are limited to invasive techniques such as micropuncture and wick-in-needle which only yield measurements at discrete locations [29, 31, 41, 42]. Indirect IFP estimates based on longitudinal monitoring of the uptake of MR contrast agents have been suggested [43–47]; however, these methods require long imaging times (2 hours in a mouse model) and at least two direct measurements of pressure to obtain quantitative IFP images [44]. In-vivo testing has shown weak or no correlation between IFP estimates derived from contrast uptake and direct measurements [45, 47, 48]. A method of accurately imaging IFP in humans would be valuable; solid tumors with high IFP could be identified, and treatments can be designed with the limiting effects of IFP in mind. IFP imaging would also be useful for monitoring the efficacy of drugs designed to lower IFP to allow sufficient doses of therapeutics to reach the tumor. Other applications include pressure related disorders such as hydrocephalus, stroke, and edema.

This work develops a numerical framework for quantitative estimation of IFP images based on a subzone-based, nonlinear inversion (NLI) MRE algorithm initially developed by Van Houten *et al*. [49]. The inversion is posed as a constrained optimization problem whose objective is to minimize the least squares difference between a set of measured displacement fields and those computed by a constitutive model. The overall problem domain is divided into a set of overlapping subzones, and the inversion is performed on the individual subzones by applying the measured displacements as Dirichlet boundary conditions on the zone surface. A significant advantage of the subzone-based approach is the opportunity for parallel computing, which substantially reduces computational time and memory storage. This method has been implemented to estimate both viscoelastic [3, 50, 51] and poroelastic [16, 24, 52, 53] material properties. Poroelastic MRE yields estimates of fluid-related quantities (such as pore-fluid pressure and hydraulic conductivity) in addition to elastic property distributions. Unfortunately, pressure boundary conditions (PBCs) needed to solve the poroelastic governing equations are generally unavailable. Previous studies assumed homogeneous type I PBCs on the exterior boundary as a simple practical approach with little physical rationale, and certainly no measurement data. In this study, the unknown PBCs are estimated from the three-dimensional full volume displacement data obtained from MRE as Neumann type (i.e., type II) by relating the fluid flow through the boundary to spatial derivatives of the displacements via the governing equations of poroelastic mechanical motion. IFP is then calculated by solving the poroelastic pressure equation for nodally distributed pore pressures through a standard finite element formulation. The new algorithm is tested on a single-inclusion numerical phantom from which synthetic displacement data is generated in the presence of added Gaussian noise.

## Methods

### Poroelastic Magnetic Resonance Elastography (MRE)

Poroelastic MRE has been described as a three-dimensional finite-element based NLI scheme that enables estimation of mechanical and hydrodynamical properties from MR measurement of displacement fields [16, 53] based on time-harmonic governing partial differential equations written in the frequency domain as

$$\begin{array}{c} \nabla \cdot \mu \nabla u+\nabla \left(\lambda +\mu \right)\left(\nabla \cdot u\right)-\left(1-\beta \right)\nabla p=-\omega^{2}\left(\rho -\beta \rho_{f}\right)u, \end{array}$$ (1a)

$$\begin{array}{c} \rho_{f}\omega^{2}\nabla \cdot \left(u\left(1-\beta \right)\right)+\nabla \cdot \left(\beta \nabla p\right)=0, \end{array}$$ (1b)

where ***u*** is the three-dimensional time-harmonic displacement vector with components *u*, *v* and *w*; *p* denotes the scalar pore-pressure field; *λ* is Lamé’s first parameter; *μ* is the shear modulus; *ρ* and *ρ*_*f*_ refer to the bulk density and pore-fluid density, respectively; and *ω* is the actuation frequency. By assuming time harmonic displacement and pressure fields, ***u*** and *p* represent the complex-valued frequency-dependent time-invariant amplitude of displacement and pore-pressure, respectively. The term *β* is related to properties of the poroelastic material including hydraulic conductivity (*κ*), porosity (*ϕ*), and apparent mass density (*ρ*_*a*_), and is given by

$$\begin{array}{c} \beta =\frac{\omega \phi^{2}\rho_{f}\kappa }{i\phi^{2}+\omega \kappa \left(\rho_{a}+\phi \rho_{f}\right)}. \end{array}$$ (2)

A more compact form of the pressure Eq (1b) can be written as [54]

$$\begin{array}{c} i\omega (\nabla \cdot u)-\nabla \cdot q=0, \end{array}$$ (3)

where ***q*** is the fluid flow and defined as

$$\begin{array}{c} q=\beta i\omega u-\frac{\beta i}{\rho_{f}\omega }\nabla p. \end{array}$$ (4)

The fluid flow is introduced to allow appropriate fluid-flow boundary conditions to be prescribed in the finite element formulation. The Dirichlet (i.e. type I) and Neumann (i.e. type II) boundary conditions are denoted as

$$\begin{array}{c} \begin{array}{ccc} & u=u_{0}\; & \text{on}\;\Gamma_{u},\\ & p=p_{0}\; & \text{on}\;\Gamma_{p},\\ & n\cdot \sigma_{E}=f_{0}\; & \text{on}\;\Gamma_{\sigma },\\ & n\cdot q=r_{0}\; & \text{on}\;\Gamma_{q}, \end{array} \end{array}$$ (5)

where $\bar{\Gamma_{u}\cup \Gamma_{\sigma }}=\bar{\Gamma_{p}\cup \Gamma_{q}}=\Gamma$; ***n*** is the unit outward normal on the overall surface, Γ, and **σ**_*E*_ is the Cauchy stress tensor defined for an isotropic, linear elastic material as

$$\begin{array}{c} \sigma_{E}=\lambda \text{tr}\left(\varepsilon \right)I+2\mu \varepsilon \;\;\text{with}\;\;\varepsilon =\frac{1}{2}\left(\nabla u+\nabla u^{T}\right). \end{array}$$ (6)

Poroelastic MRE reconstructs spatial images of mechanical and hydrodynamical properties of biphasic tissues by minimizing the least squared error between a set of measured displacement data and those computed from Eq (1) throughout the image acquisition volume. The estimated material property distribution, *θ*^⋆^, is given by

$$\begin{array}{c} \theta^{⋆}=\text{argmin}\;\Pi \left[\theta \right],\;\text{where}\;\Pi \left[\theta \right]=\frac{1}{2}\int_{\Omega }{\left(u_{c}\left(\theta \right)-u_{m}\right)}^{H}\left(u_{c}\left(\theta \right)-u_{m}\right)d\Omega , \end{array}$$ (7)

where ***u***_*c*_ and ***u***_*m*_ denote the computed displacement fields and the measured MR displacement data, respectively. The superscript *H* symbolizes the complex conjugate transpose, and *θ* represents the variables to be estimated including the shear modulus, *μ*, Lamé’s first parameter, *λ*, and the hydraulic conductivity, *κ*. Ω refers to the domain of the entire set of observations. The minimization problem is solved by iteratively updating the material property distribution, *θ*, i.e.

$$\begin{array}{c} \theta_{\text{new}}=\theta_{\text{old}}+\alpha \Delta \theta , \end{array}$$ (8)

where Δ*θ* is the ‘search direction’ to ensure reduction of the objective function and *α* is a scaling factor to promote convergence. Determination of the search direction often requires first and (or) second derivative information of the objection function with respect to *θ*. Various numerical methods such as gradient based algorithms (conjugate-gradient and quasi-Newton) [55–57] and Hessian-based algorithms (Gauss-Newton) [49, 50, 52, 54, 58] have been explored.

**Fig 1:**
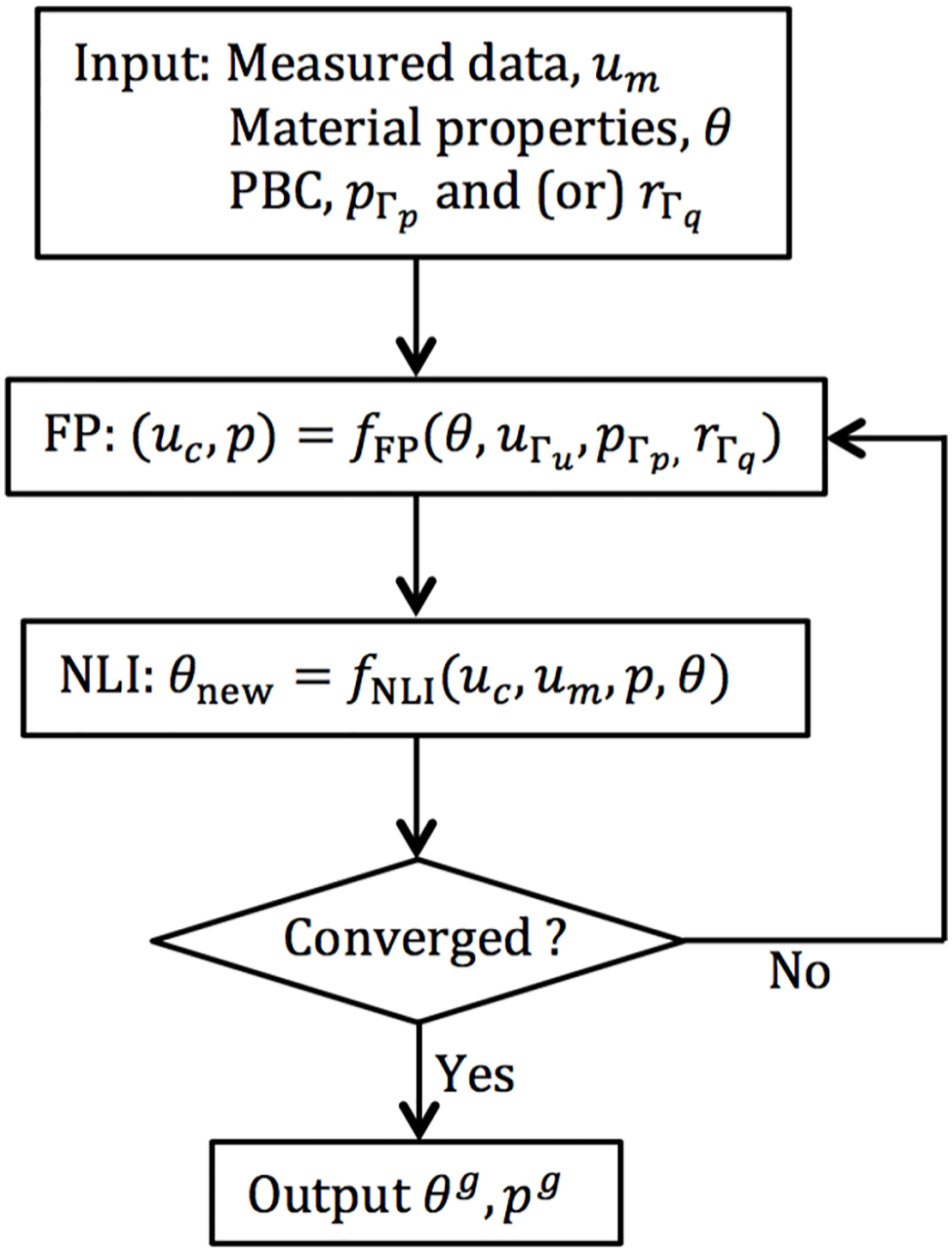
Iterative method for material property reconstruction in poroelastic MRE.

One common feature of these methods is that before updating the search direction, an estimate of the pressure field, *p*, using the current material property distribution, *θ*_old_, is needed. This step is referred to as the forward problem (FP) in elastography. The finite element method is commonly applied which produces a linear system of equations via discretization of the variational form of the poroelastic governing Eq (1) written as

$$\begin{array}{c} {}[K(\theta )]\{\begin{array}{c} u_{c}\\ p \end{array}\}=\left\{b\right\}, \end{array}$$ (9)

where ***K*** and ***b*** are the stiffness matrix and forcing vector. Once the search direction is determined, the material property distribution can be updated. This process is performed iteratively until the convergence of *θ*. A diagram showing the iterative method for material property reconstruction is presented in Fig 1. However, the algorithm suffers from two drawbacks:

1. Lack of reliable pressure measurements—For a well-conditioned forward problem, pressure boundary conditions (PBCs) need to be prescribed on the surface of the body, but physical measurements of interstitial pressure values are generally not available.
2. High computational cost—Both the FP and Δ*θ* needs to be computed repeatedly. While Δ*θ* can be calculated using subzone inversion methods, computation of *p* requires the solution of the FP. The problem size for brain has 10^4^-10^6^ unknowns; therefore, making repeated calculations for the complete imaging domain is impractical.

To address these issues, we developed a numerical framework which was built on the iterative NLI method to estimate interstitial fluid pressure (IFP) as well as the mechanical and hydrodynamical properties of poroelastic materials. The key elements of the interstitial fluid presusre imaging (IFPI) numerical framework are described in the following section.

### Interstitial Fluid Pressure Imaging (IFPI) algorithm

#### Pressure Forward Problem (PFP)

In practice, MRE measures three-dimensional displacement fields at all points in the acquisition volume. To take full advantage of the available data, we present an efficient algorithm that computes the pressure field, *p*, given the displacement data, ***u***, and the current estimate of material property distributions.

Let S and V be the trial solution and weighting function spaces, respectively, such that

$$\begin{array}{ccc} S & = & \{U=\left(u,p\right)|u_{i}\in H^{1}\left(\Omega \right),\;p\in H^{1}\left(\Omega \right),u=u_{0}\;\text{on}\;\Gamma_{u},\;p=p_{0}\;\text{on}\;\Gamma_{p}\},\\ V & = & \{W=\left(v,q\right)|v_{i}\in H^{1}\left(\Omega \right),\;q\in H^{1}\left(\Omega \right),v=0\;\text{on}\;\Gamma_{u},\;q=0\;\text{on}\;\Gamma_{p}\}, \end{array}$$ (10)

Multiplying the pressure Eq (3) with the weighting function, *q*, gives

$$\begin{array}{c} \langle \nabla \cdot q,q\rangle =\langle i\omega \nabla \cdot u,q\rangle ,\;\forall q\in V, \end{array}$$ (11)

where 〈⋅, ⋅〉 denotes the inner product over the body Ω. Using the divergence theorem, the boundary Conditions (5)_4_ and (10) result in

$$\begin{array}{c} \left\langle q,\nabla q\right\rangle =-\left\langle i\omega \nabla \cdot u,q\right\rangle +\oint_{\Gamma_{q}}r_{0}qd\Gamma_{q},\;\forall q\in V, \end{array}$$ (12)

and finally substituting Eq (4) into Eq (12) leads to

$$\begin{array}{c} \langle (\beta i\omega u-\frac{\beta i}{\rho_{f}\omega }\nabla p),\nabla q\rangle =-\left\langle i\omega \nabla \cdot u,q\right\rangle +\oint_{\Gamma_{q}}r_{0}qd\Gamma_{q},\;\forall q\in V. \end{array}$$ (13)

This system is solved using the finite element method and can be viewed as a Laplacian pressure term driven by a source involving the volumetric deformation of the solid matrix, ∇ ⋅ ***u***. Assembling terms containing the unknown pressure field, *p*, on the left side and known displacements, ***u***, on the right leads to

$$\begin{array}{c} \frac{i}{\rho_{f}\omega }\left\langle \beta \nabla p,\nabla q\right\rangle =i\omega \left\langle \nabla \cdot u,q\right\rangle +i\omega \left\langle \beta u,\nabla q\right\rangle -\oint_{\Gamma_{q}}r_{0}qd\Gamma_{q}. \end{array}$$ (14)

Note that the hydraulic conductivity, *κ*, is implicitly dependent on the parameter *β* and is spatially varying in most biological tissues; therefore, *β* is kept within the integral. The finite element discretization gives

$$\begin{array}{c} p=p_{j}\phi_{j},\;q=q_{k}\phi_{k}, \end{array}$$ (15)

where *p*_*j*_ and *q*_*k*_ are the nodally discretized description of the pressure field on the trial and weighting function spaces, respectively. A linear system can be formed to compute the nodal values of *p*, i.e.

$$\begin{array}{c} {}[K_{p}]\left\{p\right\}=\left\{b_{p}\right\}, \end{array}$$ (16)

where the **K**_*p*_ matrix contribution from the *k*’th weighting function and *j*’th interpolation function is

$$\begin{array}{c} K_{p}\left(k,j\right)=\frac{i}{\rho_{f}\omega }\langle \nabla \phi_{k},\beta \nabla \phi_{j}\rangle , \end{array}$$ (17)

and the *k*’th weighting function contributes

$$\begin{array}{c} b_{p}\left(k\right)=i\omega \langle \nabla \cdot u,\phi_{k}\rangle +i\omega \langle \beta u,\nabla \phi_{k}\rangle -\oint_{\Gamma_{q}}r_{0}\phi_{k}d\Gamma_{q}. \end{array}$$ (18)

The computational cost for PFP is lower than the full poroelastic forward problem since the size of the linear system Eq (16) is only one fourth of the size of the poroelastic forward Problem (9).

Note that the prescription of type II PBCs is not a necessary condition for a well-defined forward problem. Instead, type I PBCs need to be imposed, at least partially on the surface, to ensure the invertibility of the stiffness matrix **K**_*p*_. In the case of prescribed type I PBCs over the entire surface, the surface integral in the last term of Eq (18) is no longer required. Since no reliable measurements of the actual pressure values are available, an estimate of the type II PBC is derived from the full volume displacement data. The pressure field obtained from the PFP will be updated throughout the IFPI process as the material properties and the type II PBCs, *r*_0_, are optimized iteratively.

#### Type II Pressure Boundary Condition (PBC) estimation

From Eq (18), the PFP includes the type II PBC, *r*_0_, in addition to the displacement field, ***u***, and the hydraulic conductivity, *κ*. Here, the last term in Eq (18) is estimated from the full volume displacement data. From the weak form Eq (13) of the pressure forward equation,

$$\begin{array}{c} \oint_{\Gamma_{q}}r_{0}\phi_{k}d\Gamma_{q}=\langle (\beta i\omega u-\frac{\beta i}{\rho_{f}\omega }\nabla p),\nabla \phi_{k}\rangle +\left\langle i\omega \nabla \cdot u,\phi_{k}\right\rangle . \end{array}$$ (19)

The term ∇*p* in Eq (19) can be computed from the elasticity Eq (1a), i.e.

$$\begin{array}{c} \nabla p=\frac{1}{1-\beta }(\nabla \cdot \mu \nabla u+\nabla \left(\lambda +\mu \right)\left(\nabla \cdot u\right)+\omega^{2}\left(\rho -\beta \rho_{f}\right)u). \end{array}$$ (20)

Substituting Eq (20) into Eq (19) results in

$$\begin{array}{ccc} \oint_{\Gamma_{q}}r_{0}\phi_{k}d\Gamma_{q} & = & \langle \frac{\beta i\omega (\rho_{f}-\rho )}{\left(1-\beta \right)\rho_{f}}u,\nabla \phi_{k}\rangle +\left\langle i\omega \nabla \cdot u,\phi_{k}\right\rangle \\ & & -\langle \frac{\beta i}{\rho_{f}\omega \left(1-\beta \right)}(\nabla \cdot \mu \nabla u+\nabla (\lambda +\mu )(\nabla \cdot u)),\nabla \phi_{k}\rangle , \end{array}$$ (21)

which requires the full volume displacement field, ***u***, the hydraulic conductivity, *κ* (which is implicitly in *β*), and the elastic parameters *μ* and *λ*. Even though the elastic properties *μ* and *λ* of the solid phase do not appear explicitly in the pressure Eq (1b) (alternatively, Eq (3)), the pore-pressure field, *p*, is related to the solid matrix properties via the estimated type II pressure boundary values.

One challenging task in solving Eq (21) is the calculation of second derivative terms, ∇ ⋅ ∇***u*** and ∇(∇ ⋅ ***u***). Since linear shape functions are currently assumed in the finite element formulation, higher order derivatives of the displacement field are not defined. Therefore, multidimensional polynomial fitting [59] is applied to produce a differentiable analytical expression of the displacement as a function of (*x*, *y*, *z*). For example, a second order polynomial function defined in three-dimensional space can be expressed as

$$\begin{array}{c} f\left(x,y,z\right)=c_{0}+c_{1}x+c_{2}y+c_{3}z+c_{4}x^{2}+c_{5}y^{2}+c_{6}z^{2}+c_{7}xy+c_{8}yz+c_{9}xz, \end{array}$$ (22)

where the coefficients, *c*_*i*_ (for *i* = 1 to 9), of the polynomial regression model can be computed using linear least squares [60]. Note that the six components of the complex-valued displacement field ***u***, [i.e. Re(*u*), Im(*u*), Re(*v*), Im(*v*), Re(*w*) and Im(*w*)], are approximated individually. Higher order derivatives can then be calculated from the analytical approximation of ***u*** given in Eq (22). Furthermore, the polynomial function is smoother than the raw measurement data; thus, it can be regarded as a filtered version of both the displacement and its derivatives.

As part of algorithm development, we experimented with the highest order of polynomial terms used in (22) to represent the displacement field, and varied the range from 4 to 12. We found that if the number was too small the distribution was overly smoothed whereas if the number was too large, the approximation was overly sensitive to data noise (results not shown). We selected the highest order to be 10 as a tradeoff between over-filtering (number too low) and noisy displacement approximation (number too large), and used this polynomial representation in the algorithm to generate the outcomes shown in the **Results** section.

#### Subzone inversion

Given the size of the minimization problem in practical applications (10^4^-10^6^ unknowns), computational load must be considered. An efficient algorithm has been developed, which divides the domain Ω into a set of overlapping subzones and seeks minimization on the individual subzones with appropriate boundary values prescribed on the zone surface [49, 50, 61]. The minimization Problem (7) becomes

$$\begin{array}{c} \theta^{⋆}=\text{argmin}\;\Pi \left[\theta \right],\;\text{where}\;\Pi \left[\theta \right]=\frac{1}{2}\sum_{z=1}^{Q}\int_{\Omega_{z}}{\left(u_{c}-u_{m}\right)}^{H}\left(u_{c}-u_{m}\right)d\Omega_{z}, \end{array}$$ (23)

where Ω and Ω_*z*_ denote the domain of the total problem and a single subzone, respectively; Γ and Γ_*z*_ are the associated boundaries of Ω and Ω_*z*_. This subzone-based approach provides a natural architecture for parallel computing, as each minimization problem at the subzone level can be processed simultaneously. In the remainder of the presentation, superscript *g* refers to variables at the global level whereas those with superscript *z* refer to variables at the subzone level. Thus, the displacement field, the pressure field and the material property distribution in Ω are denoted by ***u***^*g*^, *p*^*g*^ and *θ*^*g*^. Type I and II PBCs prescribed on Γ appear as $p_{\Gamma_{p}}^{g}$ and $r_{\Gamma_{q}}^{g}$. The subzone-level variables are represented by ***u***^*z*^, *p*^*z*^, *θ*^*z*^, $p_{\Gamma_{p}}^{z}$ and $r_{\Gamma_{q}}^{z}$. Note that the prescription of $p_{\Gamma_{p}}^{g}$ is required, at least partially, on Γ in order to avoid singularity of the stiffness matrix **K**_*p*_. Here, $p_{\Gamma_{p}}^{g}$ is set to be a constant at an arbitrary boundary node as a reference value. Since only the gradient of the fluid pressure appears in the poroelastic governing Eq (1), the pressure images will be scaled by this single-node imposed type I PBC while the gradient of the pressure will not be affected. A general procedure for IFPI is based on the following steps:

1. Take $u_{m}^{g}$ from measurements, prescribe $p_{\Gamma_{p}}^{g}$, and set an initial estimate of the material property distribution, *θ*^*g*^;
2. Estimate $r_{\Gamma_{q}}^{g}$ from Eq (21) given $u_{m}^{g}$ and *θ*^*g*^;
3. Solve the PFP, i.e. Eq (16), for the global pressure field *p*^*g*^ based on $u_{m}^{g}$, *θ*^*g*^, $p_{\Gamma_{p}}^{g}$ and $r_{\Gamma_{q}}^{g}$;
4. Divide domain Ω into subzones and specify $p_{\Gamma_{p}}^{z}$ at the zone level from *p*^*g*^;
5. Solve the poroelastic FP Eq (9) for ***u***^*z*^ and *p*^*z*^ using *θ*^*z*^, $u_{\Gamma_{u}}^{z}$ and $p_{\Gamma_{p}}^{z}$, where $u_{\Gamma_{u}}^{z}$ is a type I displacement boundary condition defined by $u_{m}^{g}$;
6. Update *θ*^*z*^ via NLI.
7. Repeat steps 2-6 until convergence of *θ*.

**Fig 2:**
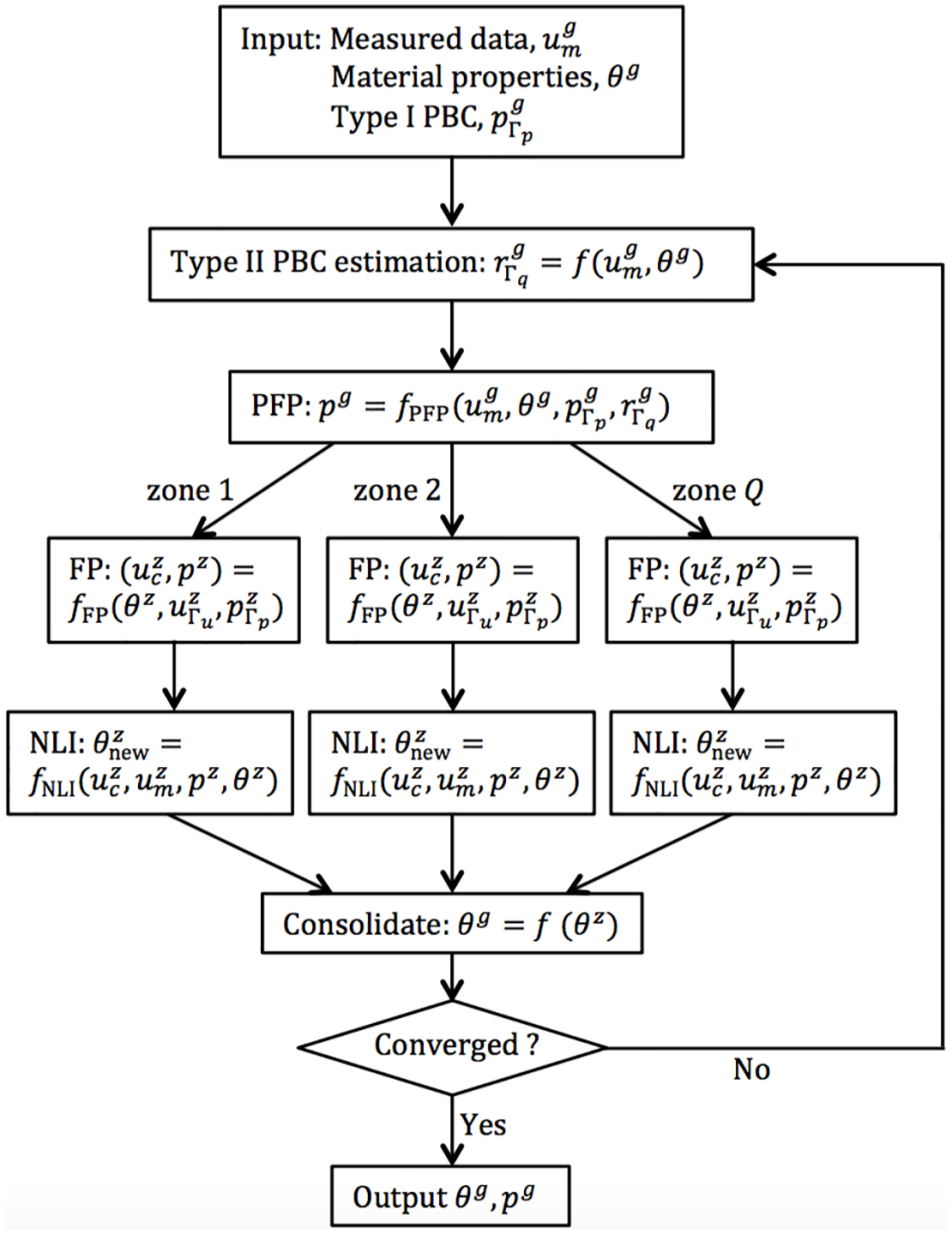
Schematic of IFPI algorithm.

A diagram illustrating the main elements of this IFPI algorithm is provided in Fig 2.

### Numerical phantom

**Fig 3:**
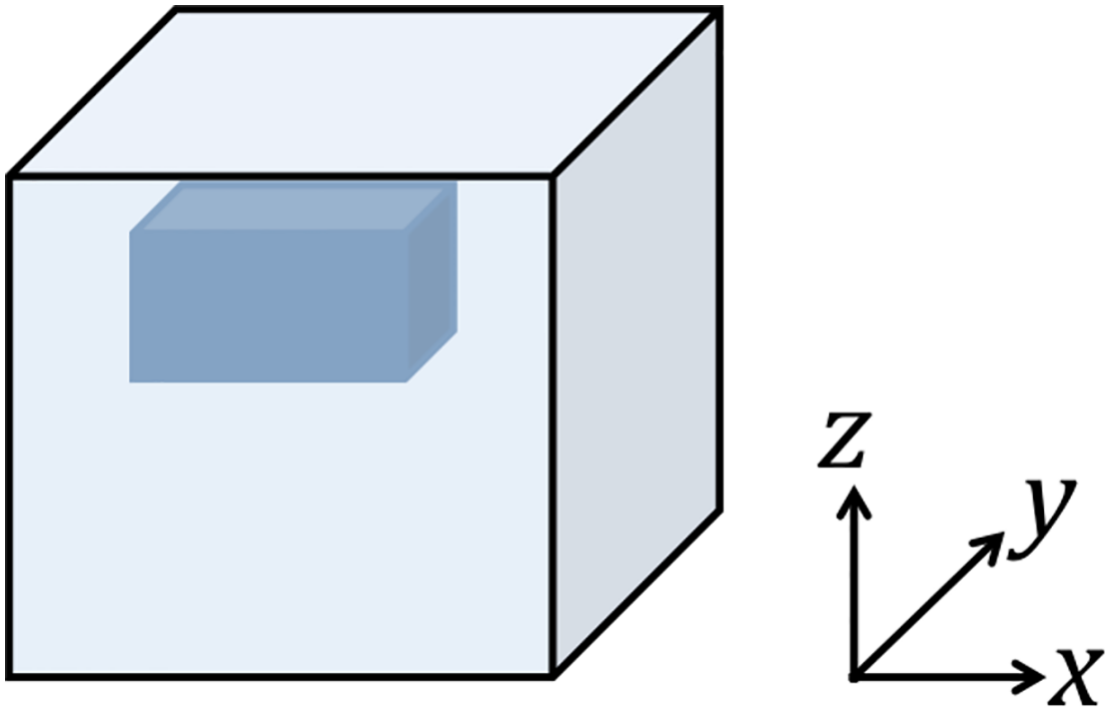
Geometry of the numerical phantom consisting of a homogeneous background and a single inclusion.

In order to validate the Fig 2 IFPI algorithm, a 6cm cube-shaped simulated phantom is considered with a single inclusion of size 3cm × 2cm × 2cm centered at *x* = 3cm, *y* = 3cm and *z* = 4.8cm. Fig 3 shows the structure of the phantom, in which the inclusion is located near the top of the cube. Displacement boundary conditions are set to be *u* = *v* = *w* = 0 (corresponding to displacements in the *x*, *y*, and *z* directions, respectively, in the Fig 3 coordinate system) on the top, *u* = *v* = 0 and *w* = 1e-2cm on the bottom of the phantom. Traction-free, i.e. ***n*** ⋅ **σ**_*E*_ = 0, boundary conditions are specified on the rest of the surfaces. The PBC is set to be *p* = 0 over the entire boundary. As shown in [62], the poroelastic model produces accurate mechanical property images at low frequencies such as those observed in intrinsic brain motion. Thus, the frequency, *ω*, is set to be 1Hz to ensure that the poroelastic model is applicable. The shear modulus, *μ*, the Lambda modulus, *λ*, and the hydraulic conductivity, *κ*, for both the inclusion and the matrix were specified based on values (ranging from 3000Pa to 6000Pa, 4500Pa to 9000Pa, and 1e-7m^3^s/kg to 1e-5m^3^/kg, respectively) observed during *in vivo* brain MRE [24]. A poroelastic global forward problem, GFP, Eq (9) is solved which computes the displacement, ***u***, and pressure, *p*, fields based on the time-harmonic poroelastic model Eq (1) with the prescribed boundary conditions and property distributions. In IFPI, the material properties and the pressure field are considered as unknowns. Computed displacements (from the GFP) act as synthetic measured data, ***u***_*m*_ (e.g., from MRE), and the algorithm described in the **Subzone Inversion** section is followed. In these numerical experiments, the type I PBC, $p_{\Gamma_{p}}^{g}$, is set to zero on a single exterior boundary node at *x* = *y* = *z* = 0, and type II PBCs, $r_{\Gamma_{q}}^{g}$, are estimated using Eq (21) at the rest of the boundary points. The resulting material property distribution, *θ*, and the pressure field, *p*, are compared with values from the GFP. To quantify errors between reconstructed estimates and original GFP solutions, a normalized root mean squared (RMS) error is tabulated, defined as

$$\begin{array}{c} \Delta f:=\frac{∥f_{\text{FP}}-f_{\text{INV}}∥}{∥f_{\text{FP}}∥},\;\text{with}\;∥\left(\cdot \right)∥:=\sqrt{\sum_{i=1}^{N}{\left(\cdot \right)}_{i}^{2}}, \end{array}$$ (24)

where *f*_FP_ and *f*_INV_ are material property or pressure values from the GFP and IFPI reconstruction, respectively.

Two cases are considered: the first reconstructs *μ* and *λ* but assumes *κ* to be known; the second estimates *κ* along with the elastic properties, *μ* and *λ*, (and are referred to as two parameter and three parameter reconstructions, respectively, in the rest of the paper and the subsections below). A simple Gaussian noise model of increasing percentage (1%, 5%, 10%, and 15%) is added directly to the GFP-derived displacement data as a way of representing uncertainty in the measurement system and testing the algorithm in the presence of noise. We did not try to model the MRI system noise from which MRE data is derived in practice. We have used the Gaussian noise approach successfully in the past when investigating new algorithms and methods associated with MRE property estimation [16, 52, 53], even though it is a simplification of reality. The lower percentages (i.e., 1%, 5%) are indicative of variability in displacement data observed in repeated measurements of the same phantom with MRE, and yield outcomes comparable to those obtained in physical experiments [53, 54]. The higher percentages (i.e., 10%, 15%) are included to explore the limits of algorithmic performance with excessively noisy data.

## Results

### Spatial derivative estimation from displacement data

**Fig 4:**
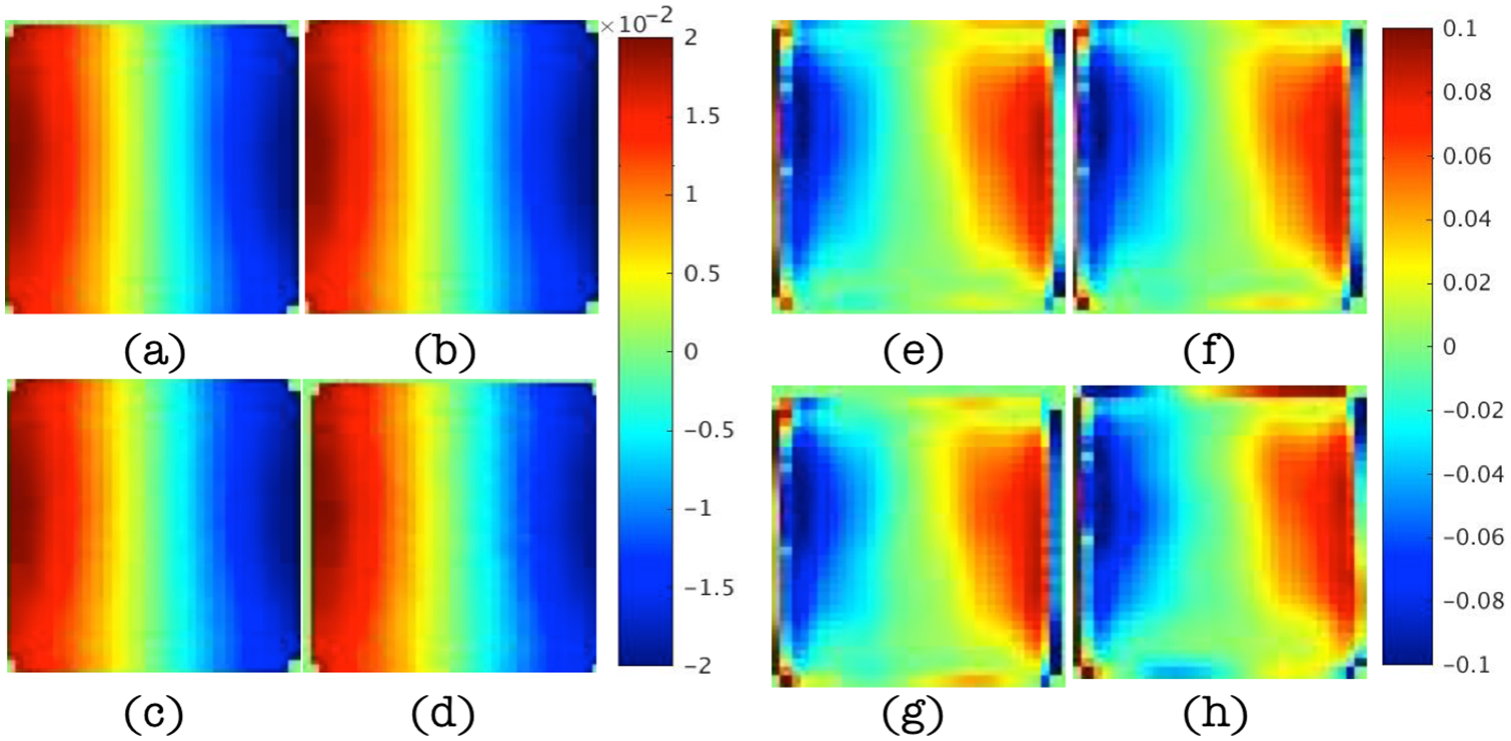
(a-d) Maps of the real displacements, *u*, in the *x* direction at slice *y* = 4.2cm for noise levels of 1%, 5%, 10% and 15%, respectively. (e-h) Corresponding maps of ∇ ⋅ ∇*u* estimated from polynomial fitting of the displacement data. Images in (a-d) are displayed in units of mm and images in (e-h) are given in units of cm^−1^.

In this subsection, results are presented to demonstrate that spatial derivatives of the displacement field are computed correctly when represented by higher order local polynomials, similar to (22), even in the presence of noise as high as 15%. Fig 4 shows the real parts of the displacement data in the *x* direction for different levels of noise and the corresponding estimations of the second order term, ∇ ⋅ ∇*u*, from multi-dimensional polynomial fitting (using polynomials with highest order 10). The magnitudes of the displacement vary from -2e-5 to 2e-5 and occur mostly along the x direction. The characteristic length scale of this variation is about one third of the cubic edge length, i.e., 2cm. Since ∂^2^ *u*/∂*x*^2^ is dominant in ∇ ⋅ ∇*u*, the magnitude of ∇ ⋅ ∇*u* can be approximated by

$$\begin{array}{c} {}[\nabla \cdot \nabla u]\approx [\frac{\partial^{2}u}{\partial x^{2}}]\approx \frac{4e-5}{0.02^{2}}=0.1, \end{array}$$ (25)

which agrees well with the polynomial-fitted results. Small distortions are found in Fig 4(g) and 4(h) when noise levels reach 10% and 15%, but in general, these higher order derivative terms are estimated effectively. The results were generated from simple compression of the numerical phantom (in the *z* direction in Fig 3) as the driving conditions (along the bottom surface as shown in Fig 3) which produced sizable components in a shearing (*x*) direction relative to the bottom surface excitation.

### Two parameter reconstruction (*μ* and *λ* estimated, *κ* known)

Material properties used in the GFP for this case are listed in Table 1. The inclusion is set to be twice as stiff as the background, while the hydraulic conductivity, *κ*, is assumed to be homogeneous throughout the domain. Displacement inputs, ***u***_*m*_, for the IFPI algorithm were obtained by solving the GFP with added noise. Finite element meshes used to compute the GFP and IFPI solutions were the same.

**Table 1:** Material properties used in two parameter reconstruction.

material property	*μ*	*λ*	*κ*
matrix	3000Pa	4500Pa	1e-7m^3^s/kg
inclusion	6000Pa	9000Pa	1e-7m^3^s/kg

**Fig 5:**
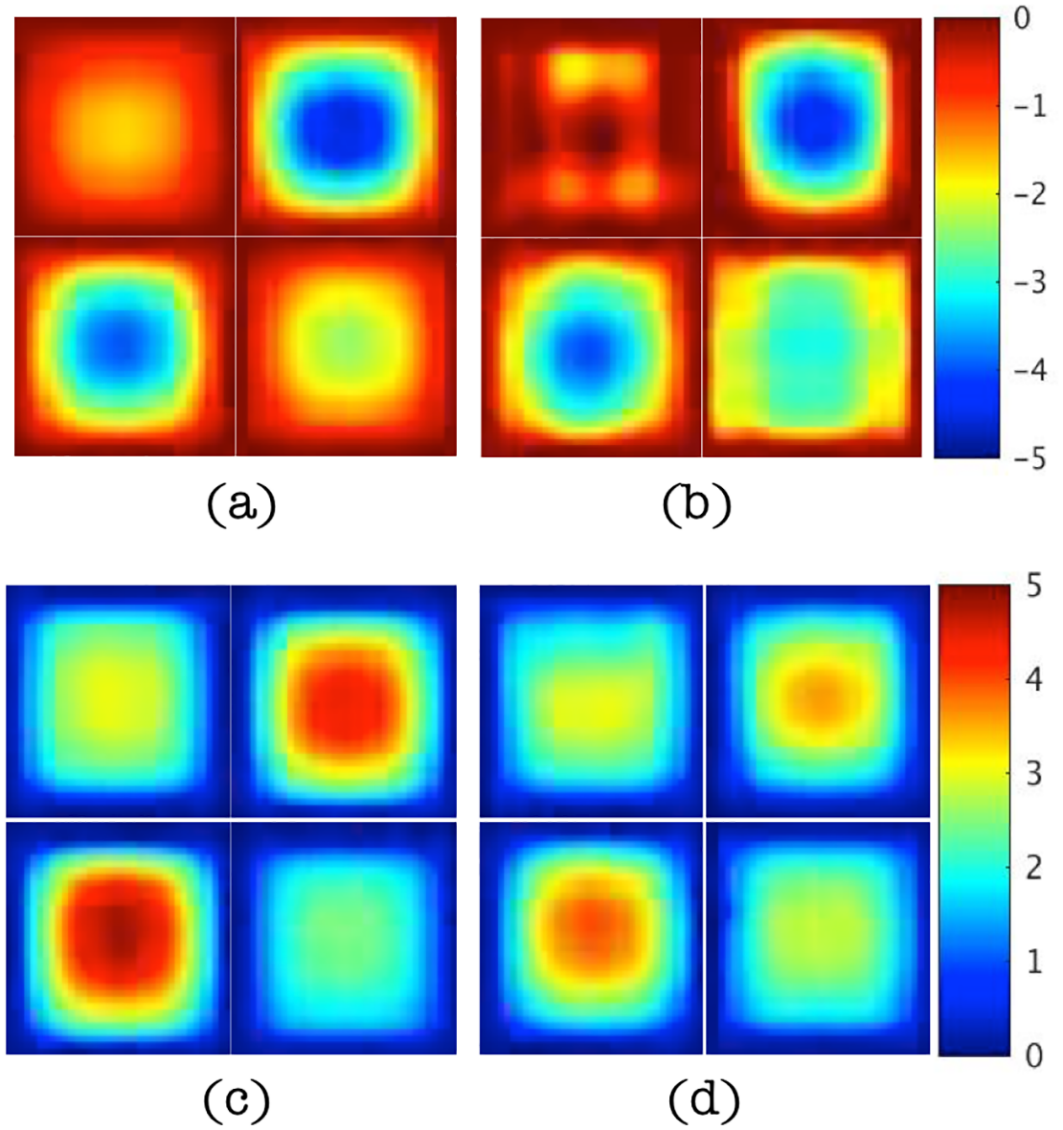
Estimated pressure, *p*, images at *y* = 0.8cm, 3cm, 4.2cm and 5.4cm in left-right clockwise orientation from 1% noisy data in the two parameter case. (a-b) Real part of the pressure field from GFP (true, left) and IFPI (estimated, right), respectively. (c-d) Imaginary part of the pressure field from GFP (true, left) and IFPI (estimated, right), respectively. Images appear in units of Pa defined by the scalebars shown.

**Fig 6:**
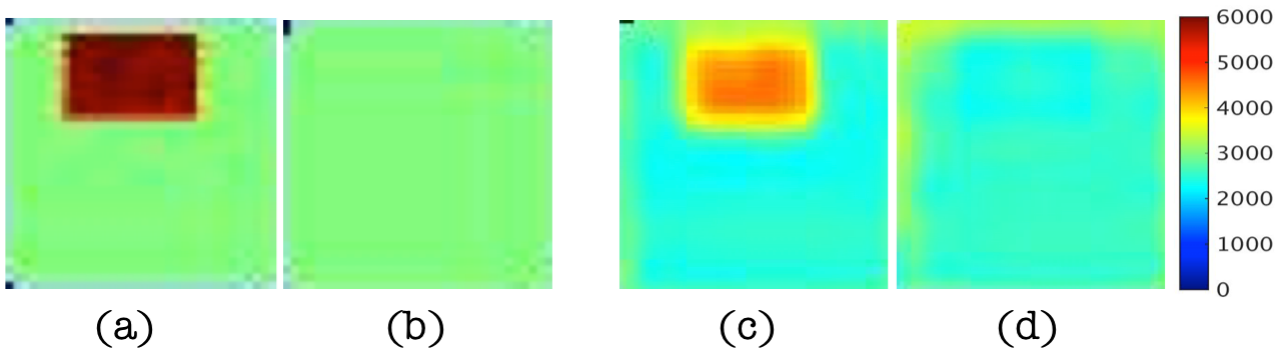
Estimated shear modulus, *μ*, images at *y* = 3cm and 5.4cm with 1% noisy data in the two parameter case. (a-b) Assigned (true) shear modulus values (left image pair). (c-d) Reconstructed shear modulus from IFPI (right image pair). Images appear in units of Pa defined by the scalebar shown.

Fig 5(a) and 5(c) present the real and imaginary parts of the pressure field, *p*, from the GFP. The corresponding pressure fields reconstructed from the IFPI algorithm (i.e. Fig 2) with 1% data noise are shown in Fig 5(b) and 5(d). Images represent *x*-*z* planes in Fig 3 at different positions of *y*. Corresponding shear modulus images for two of the four planes in Fig 5 appear in Fig 6.

**Fig 7:**
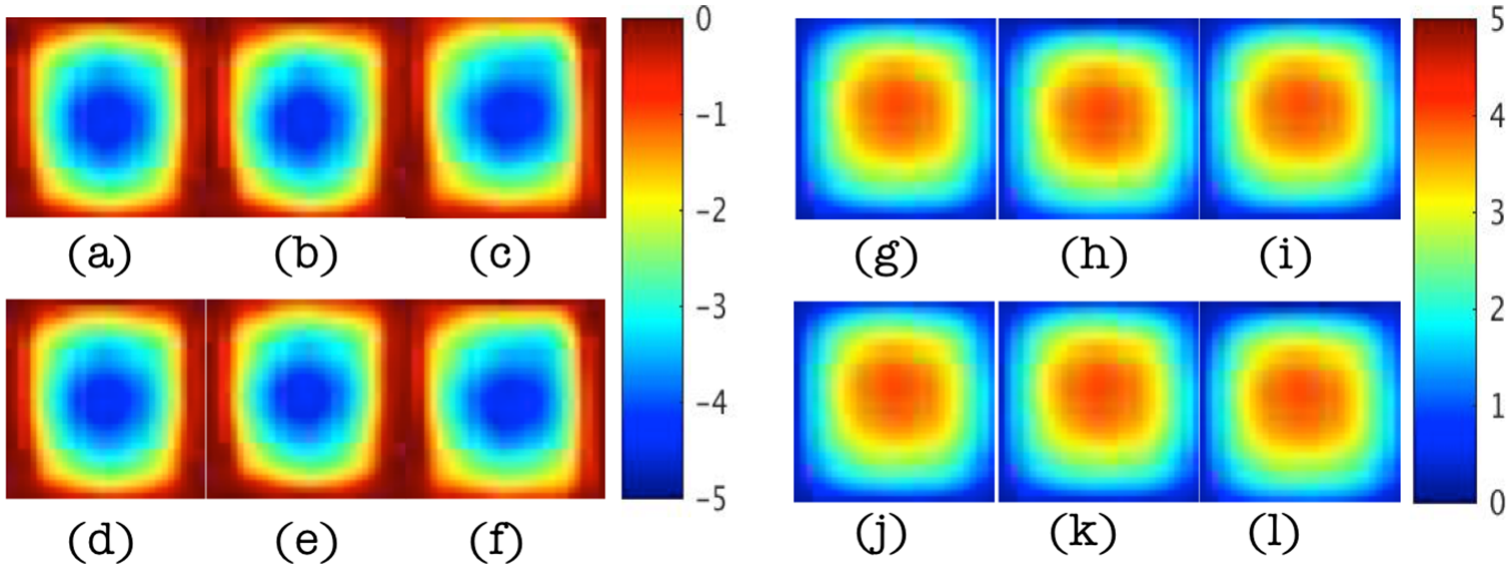
Estimated pressure, *p*, images at *y* = 3cm for noise levels of 5%, 10%, and 15% (left to right) in the two parameter case. (a-c) Real component of the pressure field estimate. (d-f) Real component of the pressure field from spatially averaged displacement data. (g-i) Imaginary component of the pressure field estimate. (j-l) Imaginary component of the pressure field estimate from spatially-averaged displacement data. Images appear in units of Pa.

**Fig 8:**
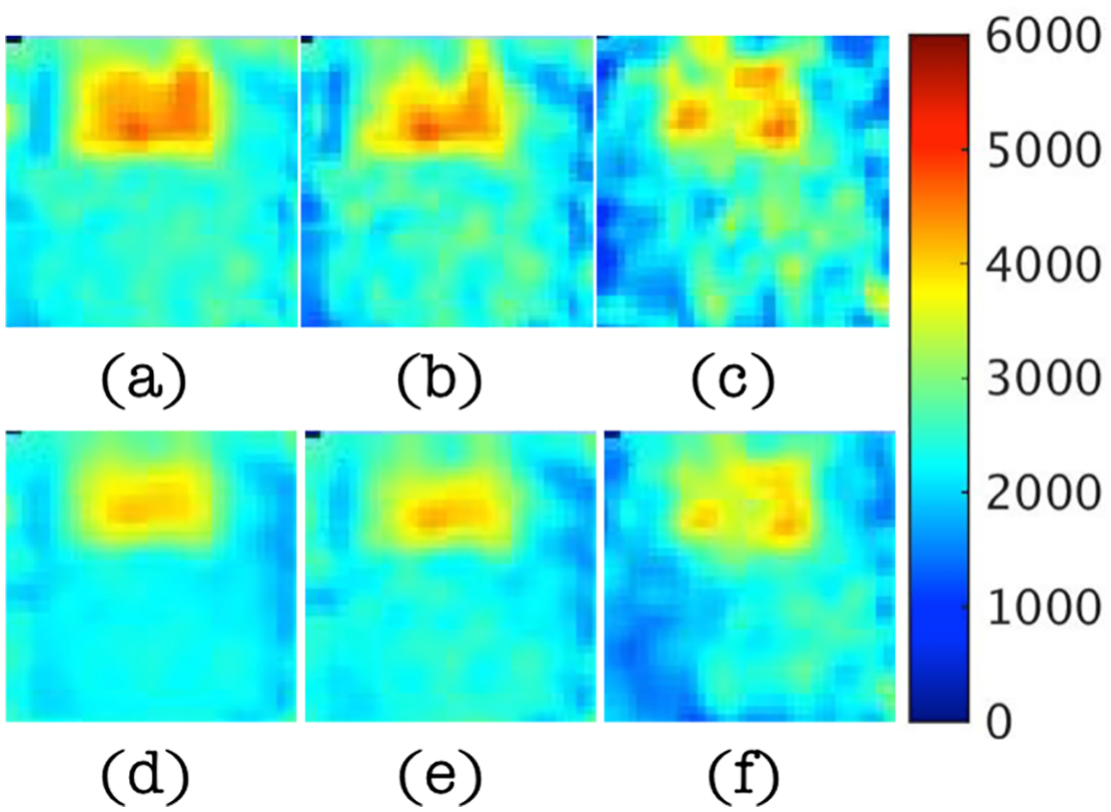
Estimated shear modulus, *μ*, images at *y* = 3cm for displacement noise levels of 5%, 10% and 15% (left to right) in the two parameter case. (a-c) Shear modulus images. (d-f) Shear modulus images from spatially-averaged displacement data. Images appear in units of Pa defined by the accompanying scalebar.

Figs 7 and 8 show reconstructed images of the estimated pressure field (Fig 7) and shear modulus distribution (Fig 8) in the *x*-*z* plane at *y* = 3cm for increasing noise levels of 5%, 10%, and 15% (true distributions appear in Fig 5(a) and 5(c), upper right image for *p*, and Fig 6(a) for *μ*). In the higher noise cases, we also averaged the displacements spatially prior to polynomial fitting and used these distributions to estimate the PBCs needed for the IPFI algorithm. IPFI results from the spatially averaged displacement data are shown in the figures as well. RMS errors in the estimated pressure field, *p*, and shear modulus, *μ*, distributions are summarized in Table 2 as a function of noise level. The reconstruction errors from the spatially averaged data are also included (images not shown in Figs 7 and 8).

**Table 2:** RMS errors in estimated quantities from the two parameter case.

noise level	1%	5%	10%	15%	〈1%〉	〈5%〉	〈10%〉	〈15%〉
*μ*	0.1024	0.2262	0.3695	0.5328	0.0945	0.1637	0.3058	0.4772
*p*	0.2184	0.2550	0.3023	0.2884	0.2178	0.2523	0.3021	0.2856

〈⋅〉 represents the spatially averaged results.

### Three parameter reconstruction(*μ*, *λ* and *κ* estimated)

In these numerical experiments, *κ* is unknown and assumed to vary spatially. As a result, it is reconstructed along with the elastic parameters, *μ* and *λ*. The material properties used in the GFP for this case are summarized in Table 3. Again, the inclusion is set to be twice as stiff as the background, but this time the hydraulic conductivity, *κ*, is also different (higher) in the inclusion relative to the background. As in the previous example, displacement inputs, ***u***_*m*_, for the IFPI algorithm were obtained by solving the GFP and adding noise to the GFP results. The finite element meshes were used to compute the GFP and IFPI solutions were the same. A similar series of figures (to those shown for the two parameter case in the previous section) is presented, starting with estimates of pressure field, shear modulus and hydraulic conductivity distributions for 1% noise followed by illustrations of the effects of increasing levels of displacement data noise on IFPI algorithm performance.

**Table 3:** Material properties used in three parameter reconstruction.

material property	*μ*	*λ*	*κ*
matrix	3000Pa	4500Pa	1e-7m^3^s/kg
inclusion	6000Pa	9000Pa	1e-5m^3^s/kg

**Fig 9:**
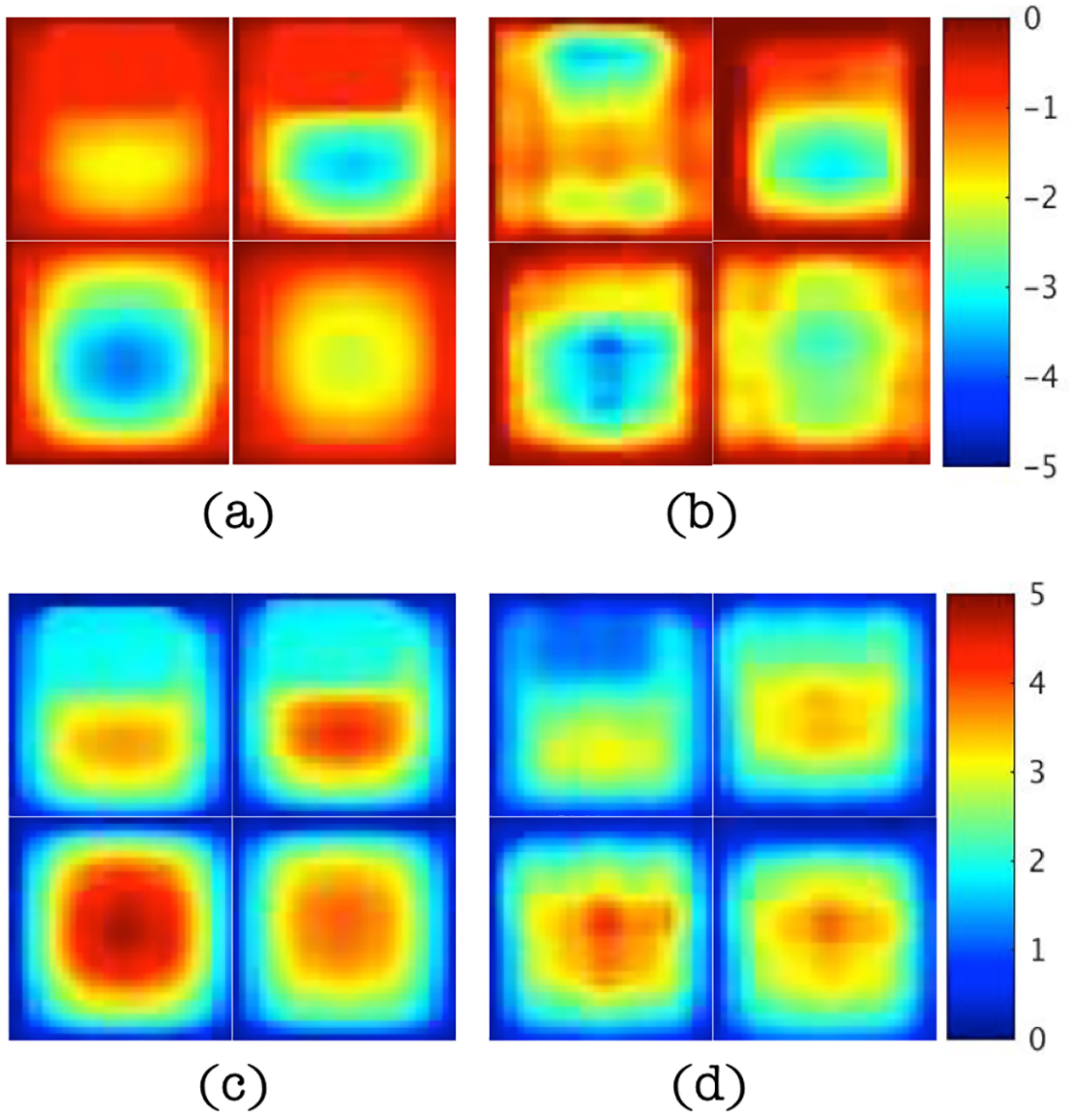
Estimated pressure, *p*, images at *y* = 0.8cm, 3cm, 4.2cm and 5.4cm in left-right clockwise orientation from 1% noisy data in the three parameter case. (a-b) Real part of the pressure field from GFP (true, left) and IFPI (estimated, right), respectively. (c-d) Imaginary part of the pressure field from GFP (true, left) and IFPI (estimated, right), respectively. Images appear in units of Pa.

**Fig 10:**
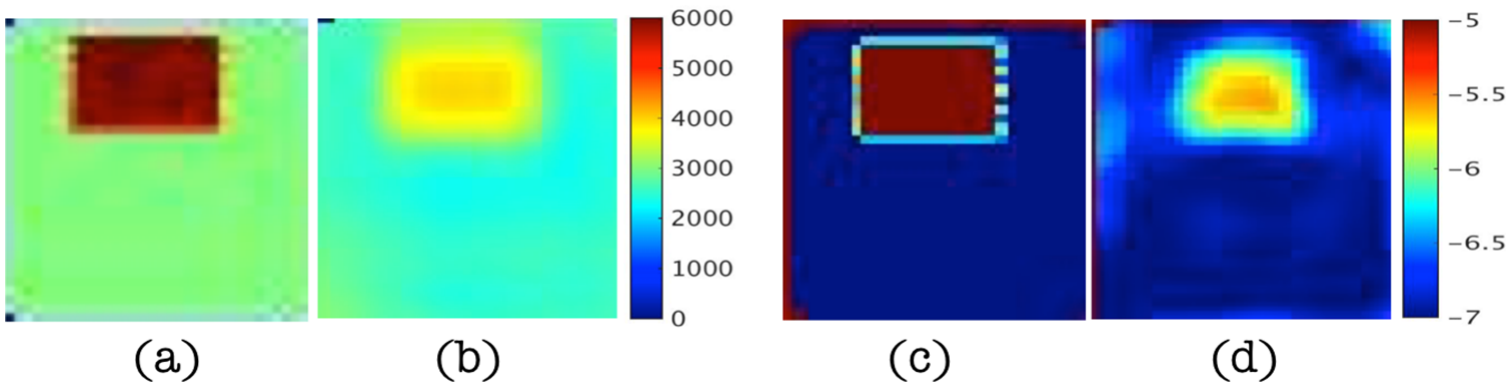
Estimated shear modulus, *μ*, and hydraulic conductivity, *κ*, images at *y* = 3cm with 1% noisy data in the three parameter case. (a-b) Assigned (true) and reconstructed shear modulus values. (c-d) Assigned and reconstructed hydraulic conductivity values from IFPI estimation. Shear modulus images appear in units of Pa whereas hydraulic conductivity images are shown in units of log_10_(m^3^/kg).

Specifically, Fig 9 shows reconstructed real and imaginary components of the pressure field relative to the true values computed with the GFP for the properties in Table 3 with 1% displacement data noise. In this case, the influence of the heterogeneous inclusion (which now has a higher *κ* value compared to the background) on the resulting pressure distribution is evident, but captured spatially and quantitatively fairly accurately (∼ 30% RMS error, see Table 4 below). Corresponding shear modulus, *μ*, and hydraulic conductivity, *κ*, images appear in Fig 10 and show similar levels of agreement, although the shear modulus image is less accurate quantitatively compared to the two parameter reconstruction case (RMS errors ∼30% vs ∼10% for two parameters), and the hydraulic conductivity is degraded further (RMS errors ∼40%). RMS errors at different noise levels (from 1% to 15%) are summarized in Table 4. They increase to more than 100% (almost 80% for pressure) for noise levels of 10% or more and the images are spatially distorted. Averaging the noisy displacement data does not offer any significant improvement.

**Table 4:** RMS errors in estimated quantities from the three parameter case.

noise level	1%	5%	10%	15%	〈1%〉	〈5%〉	〈10%〉	〈15%〉
*μ*	0.2951	0.5795	1.4321	1.7754	0.2939	0.5788	1.4968	1.7533
*p*	0.2878	0.3191	0.7781	0.7460	0.2901	0.3212	0.7850	0.7652
*κ*	0.4022	0.8542	1.9411	1.9183	0.4124	0.9441	2.378	1.9856

〈⋅〉 represents the spatially averaged results.

## Discussion

In this paper, an interstitial fluid pressure imaging (IFPI) algorithm that estimates both full field pressure distributions and material property maps from measured displacement data was developed and evaluated in a numerical phantom consisting of a background region containing an embedded inclusion with increased material property parameters. Displacement fields were generated from the numerical phantom having assigned material property parameters by solving the governing poroelastic model equations on the domain. Gaussian noise was added to the resulting displacements and the noisy values were used as synthetic data for IFPI estimation of the original pressure and material property distributions. The new IFPI approach eliminates the need for type I (Dirichlet) boundary conditions on the pressure field (which were assigned based on little physical rationale and no measurement data in the past) in favor of estimating type II (Neumann) pressure boundary conditions through the governing poroelastic equations by applying the differential operators to the measured mechanical motion. Local high-order polynomial fitting of measured displacements yielded smoothed functional forms that enabled computation of second order spatial derivatives without overwhelming noise amplification. Numerical experiments of two and three material property parameter IFPI reconstructions were evaluated.

In the two parameter case, the pressure field, *p*, was estimated along with two mechanical property parameters (shear modulus, *μ*, and Lamé’s first parameter, *λ*) while other properties (hydraulic conductivity, *κ*) were constant and assumed to be known. Under these conditions, the original type I pressure boundary conditions (*p* = 0) specified on Γ (to generate the synthetic displacement data) were recovered accurately (see Fig 5). The real and imaginary parts of the pressure field changed from zero on the surface to -5 and 5 Pa through the center of the phantom, and estimated pressures captured the correct spatial behavior but tended to underestimate their corresponding true values. Specifically, estimated pressure fields mirrored the true distributions spatially, and quantitatively, their values had RMS errors near 20% with 1% displacement noise (see Fig 5 and Table 2) that approached 30% when noise levels increased to 15% (see Fig 7).

Overall, the shape of the pressure distribution was insensitive to noise in this case, as were the underlying pressure values, although RMS errors did grow by 10% (from 20% to 30%, see Table 2) under the highest noise conditions. The pressure field’s immunity to displacement data noise results, in part, from the fact that the pressure images are relatively insensitive to the estimated shear modulus (the recovery of which is sensitive to the level of displacement data noise as disussed further below). The relative insensitivity of pressure to the estimated shear modulus is observed by substituting Eq (21) into Eq (18) which yields

$$\begin{array}{c} b_{p}\left(k\right)=\langle \frac{i\omega \beta u(\rho -\beta \rho_{f})}{\left(1-\beta \right)\rho_{f}},\nabla \phi_{k}\rangle +\langle \frac{\beta i}{\left(1-\beta \right)\rho_{f}\omega }\left(\nabla \cdot \mu \nabla u+\nabla \left(\lambda +\mu \right)\nabla \cdot u\right),\nabla \phi_{k}\rangle . \end{array}$$ (26)

Multiplying *ρ*_*f*_ *ω*/*βi* on both sides of the pressure Eq (16) leads to

$$\begin{array}{c} p\propto \frac{\omega^{2}u\left(\rho -\beta \rho_{f}\right)}{1-\beta }+\frac{1}{\beta }\left(\nabla \cdot \mu \nabla u+\nabla \left(\lambda +\mu \right)\nabla \cdot u\right), \end{array}$$ (27)

which implies that the pressure field, *p*, is proportional to the supposition of two terms. In the two parameter reconstruction tests, the first term is dominant when the hydraulic conductivity, *κ*, (implicitly in *β*) is specified, in which case, the material properties, *μ* and *λ*, appearing in the second term have less influence on the resulting pressure images.

In the two parameter case, the recovered shear modulus, *μ*, localized the heterogeneity as a stiffer inclusion with the same property difference as the assigned values (∼3000Pa) but with peaks in the inclusion and background about 1000Pa lower than the numbers in Table 1 (see Fig 6). The underestimation of material properties may result from underestimation of the type II pressure boundary conditions from the polynomial-fitted displacement data. Not surprisingly, the jump change in shear modulus at the inclusion interface was smoothed in the estimated property profile over a distance of about 3 mm which is comparable to the finite element mesh resolution. When displacement data noise increased from 1% to 5%, RMS errors in shear modulus increased from near 10% to about 20% (see Table 1). Here, the spatial distribution of shear modulus remained relatively similar, although more variation in shear modulus values did occur near the inclusion surface closest to the outer boundary of the numerical phantom (see Fig 8). Further spatial degradation of the shear modulus map, especially within the inclusion and near the outer boundaries of the numerical phantom, resulted from further increases in displacement data noise, to the point where localization of the inclusion was compromised (with 15% noise). Fortunately, high quality MRE displacement data are represented by the lower noise levels considered here (i.e, 1% and 5%), and the higher levels of noise that were evaluated are not expected in practice.

The extent to which the two parameter model and these estimation results would be clinically acceptable, or even informative, remains to be determined. Hydraulic conductivity is not well characterized as a tissue property in the biomedical literature, and as a result, the degree to which it is an important parameter that could be exploited clinically is unknown. *κ* is often assumed to be homogeneous for simplicity, and published mechanical property results estimated under these conditions appear to be reasonable at least in the normal brain [24]. Even if estimating hydraulic conductivity spatially proves to be unproductive (e.g., is not necessary, or informative, or is too difficult), questions remain about whether the RMS errors in property values observed here are tolerable. The 20%–30% error in estimated property values is high from a quantitative perspective and could limit the ability of the technique to resolve property differences on the order of these variations. No doubt smaller errors are desirable. However, spatial distributions of property change are still evident, and contrast is recovered despite the RMS errors in property values. The degree to which high accuracy in absolute property values is needed, or is important clinically, is not yet understood, and errors on the order of those reported in the studies presented here may be acceptable.

Questions on the clinical role and significance of hydraulic conductivity as a recoverable tissue property parameter motivate the three parameter case studies. When hydraulic conductivity was estimated in the reconstruction, accuracy of the pressure field recovered from IFPI decreased. Since hydraulic conductivity, *κ*, influences how easily fluid flows through the porous solid matrix [54], it has a strong effect on motion attenuation and apparent compressibility of the medium. When allowed to vary in the inclusion relative to the phantom background, *κ* distorted the pressure distribution in the neighborhood of the inclusion, and created a more complex pressure distribution as illustrated in Fig 9. With 1% displacement data noise, the IFPI algorithm recovered the spatially more complicated pressure field throughout the phantom with RMS errors approaching 30% (see Table 4). Increasing noise in the displacement data became more problematic, although pressure estimation performance was similar for 5% noise (30% RMS errors), but degraded more substantially at noise levels of 10% and 15%. Shear modulus and hydraulic conductivity estimates followed suit. They were spatially and quantitatively acceptable with 1% displacement noise (RMS errors of 30% and 40%, respectively, in Table 4), and localized the inclusion correctly, although with more spatial smoothing at the inclusion interface (jump change in material property values occurred over distances of 6 mm vs 3 mm in the two parameter case), and reduced property differences between inclusion and background relative to the assigned values. Again, these degraded property image characteristics were exacerbated by increasing levels of displacement data noise. The primary algorithmic challenge is controlling noise amplification in the estimation process, especially when more property parameters are added, in this case hydraulic conductivity, for which sensitivity to displacement data change is low. When noise is substantial in the measured data, the reconstruction algorithm alters the iterative property updates to minimize differences between calculated displacements and noisy data, which could generate unrealistic property values and distributions. Another source of error arises from using the conjugate gradient method for minimization with a single starting estimate that gets trapped in a local minimum in the high-dimensional property parameter space.

Some limitations and opportunities for improvement in the study are worth noting. First, the impact of data-model mismatch that occurs inevitably, when experimental data is acquired from materials (and tissues) which approximate poroelastic behavior, has not been considered. Here, synthetic data was generated under the ideal conditions of perfect data-model match which does not exist in practice. Nonetheless, we have found that performance observed in numerical experiments similar to those described in this paper do reflect algorithm behavior under experimental conditions, certainly in phantoms, and we would expect images comparable to those reported here, to be obtained experimentally. Second, the mechanical deformation of the numerical phantom in the test cases considered was simple and represented by uni-directional compression. More complex driving conditions will, in principle, produce more complicated displacement distributions with greater shear/compression wave interactions within and throughout the medium that could challenge the pressure boundary condition estimation from displacement data within the IFPI algorithm. Similarly, the phantom, itself, was geometrically simple and contained a single, geometrically similar inclusion. Consideration of more complex geometries and material property distributions is certainly warranted and would benefit from future studies. We have followed this approach with success during the development of similar MRE algorithms in which numerical experiments in simple test cases are conducted to explore basic algorithm behavior and are followed by more complex numerical and physical experiments in future studies. Third, algorithmic performance and noise suppression need to be improved, especially in the more challenging three parameter case. One option might be to interleave property updates with serial estimates (e.g., shear modulus then hydraulic conductivity) rather than estimating them simultaneously to improve numerical stability and sensitivity to hydraulic conductivity by first stabilizing the shear modulus estimate. Multi-start and/or global optimization methods could also diminish effects from local minima. Introducing spatial priors, for example based on MRI intensity, would down-sample and stabilize the property parameter space and could generate improved estimates, although at the risk of introducing substantial bias.

## Conclusions

Key features of this work are summarized below:

- A numerical framework for interstitial fluid pressure imaging (IFPI) of a biphasic material under time-harmonic excitation is developed, which uses displacement data from intrinsic MRE that incorporates nonlinear inversion and poroelastic modeling. A subzone approach is employed to leverage efficient parallel computation and memory storage. PBCs for the forward problem are defined as Neumann type (i.e. type II) and estimated based entirely from the full volume displacement data available from MRE. When solving the individual subzone inversion problems, type I PBCs are prescribed on the subzone surfaces by transferring the resulting global pressure field from the PFP. This additional step requires only one-fourth of the computational time needed for the full poroelastic forward problem (GFP).
- Two parameter and three parameter reconstruction experiments have been performed on a simulated single-inclusion phantom with noisy displacement data. The displacement and pressure fields were obtained by solving the full GFP with specified material property distributions, which in turn were used for validation of the inversion scheme. In addition to the elastic parameters, the hydraulic conductivity associated with the fluid phase was estimated. In the two parameter case, the material property and IFP images were accurate to within 30% in the presence of added noise up to 5% but degraded more significantly as displacement data noise reached 10% and 15%. The error rates in the three parameter case increase more dramatically, up to 58%, 85% and 32% for shear moduli, hydraulic conductivity and pressure, respectively, even at the low noise level of 5%. Some improvements in image quality were observed with spatial filtering of the noisy displacement data.
- Future studies are needed to explore the utility of the proposed algorithm in recovering the pressure distribution along with other hydrodynamical properties, first in experimental physical phantoms and then in brain tissue *in vivo*. While the numerical studies reported here demonstrate the feasibility and potential of the IFPI technique, they do not incorporate or consider the data-model mismatch that inevitably arises from the mathematical approximations of physical systems. The extent to which these errors are manageable or similar to those observed in the past warrants further investigation. Given no reliable way exists to measure IFP non-invasively, experimental studies of the IFPI algorithm are certainly worth pursuing from a variety of medical perspectives, and hopefully, will demonstrate that important information can be derived for diagnosis and treatment of cancer and pressure related diseases such as hydrocephalus.
